# COVID-19 Pandemic: Public Health Implications in Nepal

**DOI:** 10.3126/nje.v10i1.28269

**Published:** 2020-03-30

**Authors:** Mohammad Asim, Brijesh Sathian, Edwin van Teijlingen, Ahammed Mekkodathil, Supram Hosuru Subramanya, Padam Simkhada

**Affiliations:** 1 Surgery Department, Trauma Surgery, Hamad General Hospital, Doha, Qatar; 2 Centre for Midwifery, Maternal and Perinatal Health, Bournemouth University, Bournemouth, UK; 3 Manmohan Memorial Institute of Health Sciences, Nepal; 4 Nobel College, Nepal; 5 Department of Medical Microbiology, Manipal College of Medical Sciences, Pokhara, Nepal; 6 School of Human and Health Sciences, University of Huddersfield, UK

## Background

Over the past few decades, we have seen several outbreaks of zoonotic coronavirus infections. These viruses have the potential of interspecies transmission leading to pathogenesis in humans. This particular respiratory coronavirus initially named 2019-nCOV, is known as severe acute respiratory syndrome coronavirus-2 (SARS-CoV-2). Later, the World Health Organization (WHO) named this pandemic as Coronavirus disease 2019 (COVID-19). Individuals who were exposed to a wet market in Wuhan, China, were initially diagnosed with the disease. Similar to two previous outbreaks, severe acute respiratory syndrome coronavirus [SARS-CoV] and Middle East respiratory syndrome coronavirus [MERS-CoV], COVID-19 also causes respiratory illness [1]. The initial reports indicated that human-to-human transmission of this disease was limited, but soon it became evident that COVID-19 could spread through person-to-person contact more harshly in communities where there are mass gatherings [2].

Consequently, the disease was quickly transmitted to other geographical regions through infected travelers. The clinical course, the spectrum of illness, and severity of COVID-19 include three significant patterns: (1) mild illness presented with symptoms of upper respiratory tract infection; (2) non-life-threatening pneumonia; and (3) severe pneumonia manifested with acute respiratory distress syndrome which necessitates advanced life support. The most common symptoms are fever, cough and shortness of breath. The current evidence suggests that the incubation period after exposure to the COVID-19 virus is supposed to be within two weeks, and the median incubation period with symptomatic presentation appears around 5 days post-exposure [2-4].

It is known that the genome size of coronaviruses is largest among all identified RNA viruses and so these viruses are capable of recombination (homologous and non-homologous), which makes them more vulnerable for mutations. The initial genome sequence analysis of the nine patients with SARS-CoV-2 revealed 99·9% sequence identity which belongs to subgenus Sarbecovirus [5]. Recently, Zhu et al. [6] have isolated COVID-19 nucleic acid and characterized its complete genome by using high-throughput sequencing and bioinformatics. The results of initial phylogenetic analysis, together with other studies, demonstrated that the novel coronavirus has 75-80% similarity with the SARS-CoV and is also closely related to the pathogenic coronavirus infecting bats and other wild animals [7]. Furthermore, structural analysis of various COVID-19 samples from China revealed two types of SARS-CoV-2 strains designated as ‘type L,’ which constituted 70% of the strains, while the remaining 30% accounted for ‘type S’ strain [8]. During the initial outbreak in China, ‘type L’ predominated mainly in the Wuhan region, and so, this strain was less likely to be identified outside of Wuhan. The clinico-epidemiological implications of these findings necessitate further investigation. Current studies have also suggested that COVID-19 can be potentially transmitted through aerosol and fomite. The virus could remain contagious in the aerosols for up to 3 hours and remains viable on hard surfaces such as plastic and stainless steel for about 72 hours [9].

The diagnostic testing for COVID-19 includes three main strategies such as (a) detection of the virus using Reverse Transcriptase Polymerase Chain Reaction (RT-PCR) (b) detection of antibodies (IgM/IgG) to the virus using Enzyme-linked immunosorbent assay (ELISA) and (c) imaging modalities like CT scan to identify the extent of lung involvement. Among the different clinical specimens, bronchoalveolar lavage (93%) has the highest positivity rate for SARS-CoV-2 followed by sputum (72%) and nasal swabs (63%).

The experimental studies found that COVID-19 multiplies efficiently in primary human airway epithelial cells, which could be used for pre-clinical studies. Therefore, a better understanding of the novel viral characteristics and pathogenic domains allow researchers to develop rapid and highly sensitive diagnostic test and facilitate the invention of novel vaccines and antiviral therapies. Notably, the development of specific diagnostic assays will be more appropriate to assess the disease prevalence among humans and other zoonotic species responsible for the virus transmission. In the recent development for diagnostic assays, a commercial Covid-19 diagnostic (RT-PCR) test kits which is first of its kind in India has been approved by the Indian FDA / Central Drugs Standard Control Organisation (CDSCO). Finally, molecular characterization of the virus, together with bioinformatics, may aid in developing novel vaccines and antiviral therapies based on experimental studies.

## Epidemiology of COVID-19

The WHO declared COVID-19 as a pandemic on the 21st of March 2020, when a total of 183,112 cases were reported worldwide in 163 countries with 11,890 deaths [10]. China, where the first cluster of COVID-19 patients was discovered, has reported 81,394 cases and 3,295 deaths as of March 28, 2020. Italy reported most deaths outside China; 10,023 deaths out of 92,472 cases. The United States is currently leading the total number of confirmed cases worldwide. The overall mortality rate was 2.4 per million populations.When compared to China, the incidence rate of COVID-19 (1,529 vs. 57 per million population) and mortality (166 vs. 2 per million population) was considerably higher in Italy. The median age of the patients was 47 years (IQR 35-58), and nearly 60% were males [4]. The majority (81%) of the cases were mild (no or mild pneumonia); 14% were severe with dyspnea, hypoxia, or >50 percent lung involvement on imaging within 24 to 48 hours. Patients with severe disease reported to have respiratory failure, shock, or multi-organ dysfunction (5%). No deaths were reported among noncritical cases [11]. Nearly a quarter of the patients (23.7%) were having underlying comorbidities, which included hypertension (15%), diabetes (7.4%), and coronary heart disease (2.5%) [4,11]. The WHO stressed the need for immediate aggressive preventive measures to slow down COVID-19 among South-East Asia to slow down the steady increase in disease transmission. To date, the number of COVID-19 cases in South Asia has so far remained low, but there are predictions that this situation may get worst in the coming days. Collectively, the South Asian countries have around 2723 confirmed cases of COVID-19 (Pakistan 1495, India 933, Sri Lanka 113, Afghanistan 110, Bangladesh 48, Maldives 16, Bhutan 3 and five cases in Nepal) as of 28th March 2020. In India, the selective use of hydroxychloroquine for prophylaxis among high-risk healthcare professionals and caregivers has been recently approved by the Indian Council of Medical Research (ICMR).

## COVID-19 in Nepal

Nepal is among one of the nine countries that reported just five cases of COVID-19 as of March 28, 2020. Most COVID-19 cases are handled and treated at Sukraraj Tropical and Infectious Disease Hospital and the SARS-CoV-2 testing is being done at the Nepal Public Health Laboratory in Kathmandu. The first positive case of COVID-19 in Nepal was reported on January 13, 2020, in a 32-year-old Nepalese student based at Wuhan University of Technology, with no known comorbidities who had returned to Nepal [12]. The patient presented at a hospital in Kathmandu, complaining of cough. He had developed symptoms six days before his travel to Nepal. Throat swabs taken from the patient were found to be positive for COVID-19 using real-time quantification assays performed at the WHO laboratory in Hong Kong. Fortunately, the patient recovered 13 days post-exposure. The second case was confirmed on 23rd March 2020. This was a 19-year old female student who returned to Nepal from France via Qatar on March 17. The third case of Coronavirus was tested positive on 25rd march 2020 who is a 32-year-old male returned from United Arab Emirates on March 19. He stayed at a hotel in Kathmandu and later confirmed for COVID-19 . On March 27, 2020, the fourth case was test positive which is among the first confirmed case outside the Kathmandu valley. This patients is a 37-year-old male returned recently from the Middle East. The fifth case is a 19-year-old female who travelled to Kathmandu from Belgium via Qatar and was confirmed on March 28, 2020. This patient had travelled with the patient who was diagnosed on March 23 (in the same flight from Doha to Kathmandu).

Considering the risk of COVID-19 transmission, the government of Nepal has initiated various preventive measures. Several districts including Kathmandu are in lock-down where people are asked stay at home except for emergency reasons. Dedicated health-desks have been set-up at the international airport and on the border checkpoints with India and china. All foreign nationals who enter Nepal must remain in self-quarantine for 14 days and all international flights have been suspended. The government of Nepal has stopped all promotional campaigns such as ‘Visit Nepal 2020’;‘Sagarmatha Sambad 2020’ and others international events.

Having only five cases of COVID-19 in Nepal is, of course, unrealistic, especially considering the high influx of Chinese either as a tourist or as a worker, through several border crossing points, and unrestricted migration of tourists from countries with severe outbreak mainly from South Korea, Italy, Spain, Japan, and France till 9th of March 2020. The low incidence may reflect improper diagnoses due to the absence of testing. We are still in the early stage of the pandemic, so infection and mortality rates are bound to rise considerably. Therefore, it is a crucial time for governments to implement stringent measures to control the spread further.

## Challenges to contain COVID-19 spread in Nepal

There are various public health challenges in controlling the spread of COVID-19 in South Asian region, including Nepal. Learning from the COVID-19 outbreak in China, there will be a slowdown of economic activity with damaged supply chains that impact the public health systems in Nepal. Moreover, there is limited coordination among different stakeholders in healthcare management with few policies in place for infection prevention and control (IPC), shortage of testing kits, medical supplies, personal protective equipment, and poor reporting are major challenges to be tackled in case of the COVID-19. Most South Asian countries are vulnerable to a mass outbreak owing to high population density in cities, where it is more challenging to maintain social distancing, poor hygiene and lower (health) literacy rate. Additionally, some COVID-19 cases remain asymptomatic, so it is difficult to predict the severity of the outbreak.

## Recommendations

Currently, there is a need to allocate resources, manpower and stricter regulations to contain the COVID-19. The WHO has published a set of preventive guidelines on COVID-19 for health workers [13]. Importantly, the healthcare staff should be trained to tackle complex situations and challenges associated with the disease outbreak and to implement strict IPC measures to avoid transmission. The intensive awareness campaign for surveillance, quarantine, and diagnosis should be rolled out. Additionally, there is a need to focus on guidelines, education, and training for IPC through multifaceted strategies, regular monitoring, review of practices and feedback, staffing, and bed occupancy in acute healthcare settings.

The management for COVID-19 primarily comprized of supportive care. So, home management may be possible for patients with mild illness who should be properly isolated. To futher reduce the risk of transmission in the community, individuals should maintain hand hygiene, avoid touching eyes, nose, and mouth, cover the face while coughing or sneezing and avoid close contact with sick individuals. Due to short supply, face masks (preferably N95) are only advised for healthcare professionals and individuals with symptoms of respiratory distress. Unless appropriately worn, the face masks are ineffective for preventing the disease exposure. Social distancing is advised in locations that are at higher risk of community transmission, as one should keep at least 1½ to 2 meters distance from another person.

In the current situation, abiding the general health guidelines is utmost important to maintain stronger immunity to fight infection and protection from other environmental hazards. The healthy lifestyle practice includes good eating habits, regular exercise, control of stress and blood pressure level, avoidance of smoking and drinking alcohol and get enough sleep. However, this means Nepal also needs to address health inequality and poverty which are existing public health issues in the country. Poor people have fewer opportunities to eat well, maintain hygeine and keep social distance from ill. Finally, collaboration between national agencies in Nepal with international organizations such as the CDC and WHO [13] can help to contain the spread of COVID-19 through global best practices.
